# Comparison of molecular testing strategies for COVID-19 control: a mathematical modelling study

**DOI:** 10.1016/S1473-3099(20)30630-7

**Published:** 2020-12

**Authors:** Nicholas C Grassly, Margarita Pons-Salort, Edward P K Parker, Peter J White, Neil M Ferguson, Imperial College COVID-19 Response Team, Kylie Ainslie, Marc Baguelin, Samir Bhatt, Adhiratha Boonyasiri, Nick Brazeau, Lorenzo Cattarino, Helen Coupland, Zulma Cucunuba, Gina Cuomo-Dannenburg, Amy Dighe, Christl Donnelly, Sabine L van Elsland, Richard FitzJohn, Seth Flaxman, Keith Fraser, Katy Gaythorpe, Will Green, Arran Hamlet, Wes Hinsley, Natsuko Imai, Edward Knock, Daniel Laydon, Thomas Mellan, Swapnil Mishra, Gemma Nedjati-Gilani, Pierre Nouvellet, Lucy Okell, Manon Ragonnet-Cronin, Hayley A Thompson, H. Juliette T Unwin, Michaela Vollmer, Erik Volz, Caroline Walters, Yuanrong Wang, Oliver J Watson, Charles Whittaker, Lilith Whittles, Xiaoyue Xi

**Affiliations:** a MRC Centre for Global Infectious Disease Analysis, Department of Infectious Disease Epidemiology, Imperial College London, London, UK; b The Vaccine Centre, Department of Clinical Research, Faculty of Infectious and Tropical Diseases, London School of Hygiene and Tropical Medicine, London, UK

## Abstract

**Background:**

WHO has called for increased testing in response to the COVID-19 pandemic, but countries have taken different approaches and the effectiveness of alternative strategies is unknown. We aimed to investigate the potential impact of different testing and isolation strategies on transmission of severe acute respiratory syndrome coronavirus 2 (SARS-CoV-2).

**Methods:**

We developed a mathematical model of SARS-CoV-2 transmission based on infectiousness and PCR test sensitivity over time since infection. We estimated the reduction in the effective reproduction number (*R*) achieved by testing and isolating symptomatic individuals, regular screening of high-risk groups irrespective of symptoms, and quarantine of contacts of laboratory-confirmed cases identified through test-and-trace protocols. The expected effectiveness of different testing strategies was defined as the percentage reduction in *R*. We reviewed data on the performance of antibody tests reported by the Foundation for Innovative New Diagnostics and examined their implications for the use of so-called immunity passports.

**Findings:**

If all individuals with symptoms compatible with COVID-19 self-isolated and self-isolation was 100% effective in reducing onwards transmission, self-isolation of symptomatic individuals would result in a reduction in *R* of 47% (95% uncertainty interval [UI] 32–55). PCR testing to identify SARS-CoV-2 infection soon after symptom onset could reduce the number of individuals needing to self-isolate, but would also reduce the effectiveness of self-isolation (around 10% would be false negatives). Weekly screening of health-care workers and other high-risk groups irrespective of symptoms by use of PCR testing is estimated to reduce their contribution to SARS-CoV-2 transmission by 23% (95% UI 16–40), on top of reductions achieved by self-isolation following symptoms, assuming results are available at 24 h. The effectiveness of test and trace depends strongly on coverage and the timeliness of contact tracing, potentially reducing *R* by 26% (95% UI 14–35) on top of reductions achieved by self-isolation following symptoms, if 80% of cases and contacts are identified and there is immediate testing following symptom onset and quarantine of contacts within 24 h. Among currently available antibody tests, performance has been highly variable, with specificity around 90% or lower for rapid diagnostic tests and 95–99% for laboratory-based ELISA and chemiluminescent assays.

**Interpretation:**

Molecular testing can play an important role in prevention of SARS-CoV-2 transmission, especially among health-care workers and other high-risk groups, but no single strategy will reduce *R* below 1 at current levels of population immunity. Immunity passports based on antibody tests or tests for infection face substantial technical, legal, and ethical challenges.

**Funding:**

UK Medical Research Council.

## Introduction

Different countries have taken very different approaches to molecular testing in response to the COVID-19 pandemic. The observation that countries with high rates of testing for severe acute respiratory syndrome coronavirus 2 (SARS-CoV-2) infection have effectively controlled transmission (eg, South Korea and Germany) has led to calls for increased testing in other countries with lower rates of testing (eg, the UK and the USA). However, the contribution of testing to COVID-19 control compared with other interventions such as self-isolation and physical distancing is currently unclear.

There is a clear priority to test patients with suspected COVID-19 in hospital to inform treatment and infection control strategies, and to monitor the extent of the pandemic. As testing capacity increases, the role of wider testing in different risk groups should be determined, not only to monitor the pandemic but also to prevent transmission by isolating infected individuals. Targets for testing could include health-care and social-care workers, care home residents, other high-risk groups, or the general population.

Health-care workers have been disproportionately affected by COVID-19, constituting between 5% and 19% of all reported COVID-19 cases in European countries (eg, from 5·1% in the UK^1^ to 19% in Spain^2^). This high incidence (six-fold higher than in the general population in the UK^3^) reflects their exposure to SARS-CoV-2 infection from patients and fellow staff. Infection in health-care workers can contribute to nosocomial spread of SARS-CoV-2, and similar concerns apply to transmission among care home staff and others working with vulnerable populations. Regular screening of these high-risk groups for infection, in addition to routine testing of those with COVID-19 symptoms, could identify individuals with mild or asymptomatic infection and reduce SARS-CoV-2 transmission if they self-isolate.

**Research in context**

**Evidence before this study**

Evidence on the performance of severe acute respiratory syndrome coronavirus 2 (SARS-CoV-2) tests has been accumulating, and our understanding of COVID-19 epidemiology has been evolving rapidly, with much of the scientific evidence still only available as preprints rather than peer-reviewed publications. We therefore searched PubMed and the medRxiv preprint server (using the R package medrxivr) using the search term “(SARS or COVID or coronavirus) and test* and model and math*” on June 1, 2020, for papers published or uploaded in 2020. The search returned 20 publications and 43 preprints, from which we identified 11 studies that used mathematical modelling to evaluate the role of testing in COVID-19 control, including two preprints missed by the search but known to the authors. Two studies examined regular screening of health-care workers for infection, one considered the effectiveness of test-and-trace strategies (assuming 100% test sensitivity), three studies examined universal mass testing (irrespective of symptoms), and six modelled increased testing simply as an increase in the rate at which infections were detected and isolated. Model results were often divergent, reflecting different assumptions about SARS-CoV-2 epidemiology and testing capacity. Weekly screening of health-care workers was estimated to prevent between 5% and 24% of transmission from this group, test and trace reduced the reproduction number (*R*) for SARS-CoV-2 transmission between 15% and 50% depending on coverage and timeliness of test results, and estimates for the effectiveness of universal mass testing varied, from a 2% reduction in *R* to a 40% reduction in epidemic size, depending on coverage and frequency. Very few studies considered test performance (sensitivity and specificity) and only one study considered more than one testing strategy.

**Added value of this study**

Our study evaluates the optimal use of available SARS-CoV-2 nucleic acid and antibody tests for prevention of transmission. We developed a mathematical framework describing infectiousness and test sensitivity over time since infection to estimate the effectiveness of alternative strategies, including regular screening of high-risk groups such as health-care and social-care workers and the effectiveness and efficiency of test-and-trace protocols. We integrated current evidence for the sensitivity and specificity of available tests alongside epidemiological data about the proportion of asymptomatic infections and the contribution of individuals with asymptomatic and presymptomatic infections to transmission. We did sensitivity analyses to determine the robustness of our results and considered the implications of our findings for national testing policies.

**Implications of all the available evidence**

Testing can play an important role in the prevention of SARS-CoV-2 transmission, in addition to its established use for pandemic surveillance and confirmation of a COVID-19 diagnosis. Optimal strategies should include regular screening of high-risk groups such as health-care and social-care workers during periods of sustained transmission, and testing of people with COVID-19 symptoms and tracing and quarantining their contacts. Test and trace requires high coverage (proportion of cases tested and contacts successfully traced and quarantined) and rapid testing and contact tracing to be effective. Testing alone is unlikely to bring *R* below 1 at current levels of immunity and will need to be complemented by other interventions such as physical distancing.

Testing could also contribute to prevention of SARS-CoV-2 transmission in the general population. It might promote adherence to self-isolation by individuals showing COVID-19 symptoms who test positive. It is also the basis of test-and-trace interventions, which involve tracing and quarantining contacts of laboratory-confirmed cases.

Mathematical models can be used to evaluate the potential effectiveness of different testing strategies. A small number of modelling studies have examined the role of regular screening in the general population or health-care workers and the role of testing during contact tracing.^4^, ^5^, ^6^, ^7^ However, these studies have assumed 100% accuracy of available tests or that testing simply increases the rate at which infected individuals are isolated.

We aimed to investigate the potential impact on SARS-CoV-2 transmission of alternative strategies for deploying PCR tests that identify active infection and antibody tests that indicate past infection. We developed a mathematical model of transmission to estimate the effectiveness of strategies aimed at health-care workers, other high-risk groups, and the general population, including regular testing irrespective of symptoms and test-and-trace strategies. We also explored the concept of so-called immunity passports, which have been proposed as an approach to certify individuals with evidence of past infection or acquired immunity as safe to return to work where there may be risk of exposure to SARS-CoV-2. We focus on the UK, but our results are relevant for policy decisions made by other countries.

## Methods

### Mathematical model

We developed a mathematical model of SARS-CoV-2 transmission, describing infectiousness over time since infection based on observed serial intervals.^8^ We used this model to evaluate the impact of self-isolation following either a positive test result or symptom onset, and the impact of quarantine of contacts of laboratory-confirmed cases. We assumed a proportion of infections are asymptomatic and that individuals who are asymptomatic might have lower infectiousness than those with symptomatic infections. We derived formulae for the effective reproduction number (*R*), defined as the average number of secondary infections from a single infected individual, in a population with testing of individuals with symptomatic infection only, screening of all individuals irrespective of symptoms, and test and trace, where contacts of laboratory-confirmed cases are quarantined. We calculated the expected effectiveness of different testing strategies, defined as the percentage reduction in *R*. We did sensitivity analyses of these model estimates to uncertainty in model parameters using bivariate plots and Latin Hypercube Sampling from model parameters with 1000 replicates to generate 95% uncertainty intervals (95% UIs). The model is described in detail in the appendix (pp 2–4) and the full code is online.

### Model parameters and data

We obtained a best estimate and plausible range for model parameters describing the natural history and transmission characteristics of SARS-CoV-2 from the published literature (appendix p 5). These included the proportion of infections that are asymptomatic and their relative infectiousness, the mean serial interval in the absence of self-isolation, the incubation period distribution, and PCR test sensitivity over time since infection. We generated an estimate of the PCR test sensitivity from three published meta-analyses of data collected after symptom onset and an assumption that presymptomatic sensitivity was proportional to infectiousness (appendix p 6).^9^, ^10^, ^11^ Specificity of PCR was assumed to be 100% on the basis of the performance of the majority of available tests reported by the Foundation for Innovative New Diagnostics.^12^ We extracted data on the sensitivity and specificity of IgG (or IgG plus either IgM or IgA) antibody tests based on tests of samples from PCR-positive COVID-19 cases in the same Foundation for Innovative New Diagnostics database accessed on June 3, 2020.^12^ We used these data to determine the positive predictive value of a positive antibody test that might be used to issue an immunity passport.

### Role of the funding source

The sponsor of the study had no role in study design, data collection, data analysis, data interpretation, or writing of the report. The corresponding author had full access to all the data in the study and had final responsibility for the decision to submit for publication.

## Results

**Figure 1:**
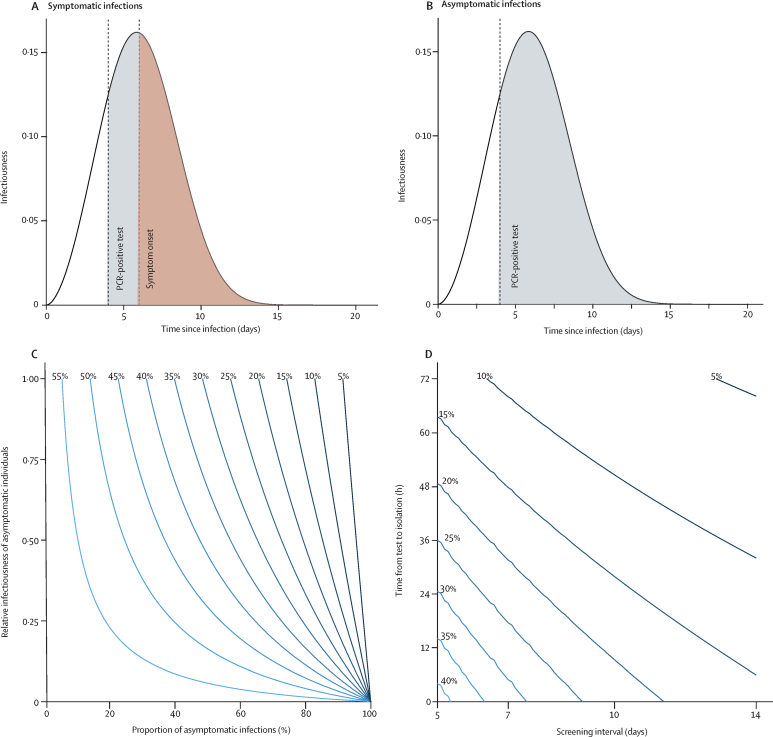
Infectiousness of SARS-CoV-2 over time since infection estimated from the serial interval and the reduction of transmission as a result of self-isolation after symptoms or a PCR-positive test result (A) Detection of presymptomatic SARS-CoV-2 infection and subsequent reduction in transmission through self-isolation after a positive PCR test. (B) Detection of asymptomatic SARS-CoV-2 infection and subsequent reduction in transmission through self-isolation after a positive PCR test. The shaded areas in these plots illustrate infectiousness that would be limited by PCR testing (grey) at 4 days after infection or self-isolation following symptom onset (red) at 6 days after infection. The area under the curves is equal to the reproduction number. (C) Percentage reduction in the reproduction number by self-isolation following onset of symptoms as a function of the proportion of infections that are asymptomatic and their relative infectiousness compared with symptomatic infections. (D) Additional percentage reduction in the reproduction number by a policy of repeated PCR testing at regular intervals with different timeliness from sample collection to isolation, assuming a third of infections are asymptomatic and that they are 50% as infectious as symptomatic infections. SARS-CoV-2=severe acute respiratory syndrome coronavirus 2.

If individuals self-isolate following onset of symptoms of COVID-19 (cough or fever or loss of smell or taste) then their contribution to SARS-CoV-2 transmission in the community will be reduced (figure 1A). The overall reduction in *R* in a population that undergoes symptom-based self-isolation will depend on the proportion of infections that are asymptomatic and their relative infectiousness compared with symptomatic infections. Current estimates indicate that approximately 33% (range 20–50) of infections are asymptomatic, and asymptomatic infections have a relative infectiousness of about 50% (range 10–100; appendix p 5). If all individuals with symptoms compatible with COVID-19 self-isolated and there was no further transmission from self-isolating individuals, this would result in a reduction in *R* of 47% (95% UI 32–55; figure 1C). The effectiveness of self-isolation also depends on the extent of presymptomatic transmission. Increasing the median serial interval from 6 days to 8 days (in the absence of self-isolation) decreases the proportion of SARS-CoV-2 transmission from presymptomatic individuals (from 42% to 26%) and results in a corresponding increase in the effectiveness of self-isolation following symptom onset from 47% to 60%. The effectiveness of self-isolation also scales linearly with compliance and the ability of self-isolating individuals to restrict transmission to other household members (eg, if self-isolation meant SARS-CoV-2 transmission was only reduced by 50% after symptom onset, *R* would be reduced by 23% instead of 47%).

PCR testing of symptomatic individuals to identify SARS-CoV-2 infection would reduce the number of individuals needing to self-isolate. However, it would also reduce the effectiveness of self-isolation because some test results would be false negatives. If samples were collected close to the time of symptom onset and test results were made available rapidly (eg, within 24 h), then the proportion of false-negative results would be relatively low (around 10%) on the basis of the reported PCR test sensitivity, and most symptomatic individuals could safely return to work once well. For example, with a 1% prevalence of SARS-CoV-2 infection, a single negative test would have a greater than 99% probability of being correct (negative predictive value). A negative test would also release any quarantined household members who might be required to stay at home (eg, in the UK, cohabitants of someone with COVID-19 symptoms currently need to quarantine for 14 days).

Regular PCR testing of high-risk groups such as health-care or social-care workers for SARS-CoV-2 infection, irrespective of symptoms, could further reduce transmission if asymptomatic or presymptomatic infections are identified and isolated (figure 1A, B). The effectiveness of this strategy depends on the frequency of testing, timeliness of results, and sensitivity of the test as a function of time since infection. For our best estimate of test sensitivity (appendix p 6), weekly screening of health-care workers and a 24 h delay from testing to self-isolation would reduce their contribution to SARS-CoV-2 transmission (*R*) by 23% on top of any reductions already achieved as a result of self-isolation following symptoms (figure 1D). If tests were to be done at the end of a shift and results made available before the next shift, then the time delay between testing and isolation would effectively be zero, increasing the effectiveness of PCR testing to 32% (depending on exposure during time off).

The effectiveness of regular screening also depends on the proportion of infections that are asymptomatic and their relative infectiousness as well as the median serial interval (in the absence of self-isolation; appendix p 7). Accounting for uncertainty in these parameters by use of Latin Hypercube Sampling gives a 95% UI of 16–40 for the effectiveness of weekly screening with a 24 h delay and 24–50 when there is no delay. Lower test sensitivity reduces the effectiveness of regular screening. For example, a 15% reduction in test sensitivity reduces the effectiveness of weekly screening from 23% (95% UI 16–40) to 19% (13–35; appendix p 7).

**Figure 2:**
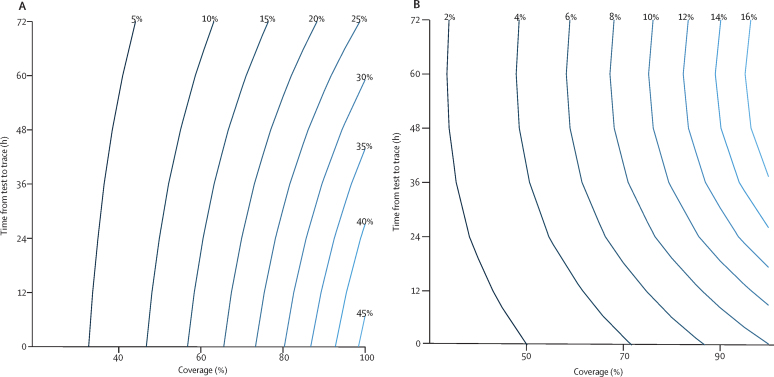
Effectiveness of test-and-trace strategies as a function of the time from test to quarantine of contacts and coverage Coverage is defined as the proportion of symptomatic infections identified for contact tracing and the proportion of contacts successfully traced, assumed equal in these plots. The percentage reduction in the reproduction number of contacts for test-and-trace strategies (A) or test-trace-test strategies (B), assuming self-isolation on the basis of symptoms is already in place. This reduction is approximately equal to the reduction in the overall reproduction number if contacts who develop COVID-19 symptoms are also eligible to be an index case for further contact tracing.

Quarantining the contacts of symptomatic individuals who test positive for SARS-CoV-2 has the potential to prevent transmission from both symptomatic and asymptomatic infected contacts. The effectiveness of this test-and-trace strategy is strongly dependent on the proportion of symptomatic individuals who are tested (*u*), the success in tracing their contacts (*v*), and the timeliness of obtaining test results and identifying and quarantining contacts. If we assume the coverage of test and trace is 80% (*u*=*v*=80%) and that it takes 24 h from sample collection to quarantine of contacts, then the reduction in the number of secondary infections from contacts of the index case is 26% (95% UI 14–35; figure 2A). If symptomatic contacts are also eligible for contact tracing, then this reduction is approximately equal to the overall reduction in *R* (see appendix pp 2–4 for further details). The combined impact of quarantine of contacts and self-isolation of symptomatic individuals is therefore a 61% reduction (95% UI 44–77) in *R* for these parameter values. On average, quarantine of contacts would occur 3·5 days after they became infected (or 2·5 days if there was no delay from sample collection to quarantine). For 50% coverage and a 48 h delay to quarantine, the reduction in transmission is just 8% (95% UI 5–11). Uncertainty intervals account for uncertainty in the proportion of infections that are asymptomatic and their relative infectiousness, the median serial interval, and test sensitivity (see appendix p 8 for further details). We assume sample collection for testing is done at symptom onset. Later testing will further reduce the effectiveness of this strategy.

Contact tracing could be done on the basis of symptoms alone, rather than waiting for a test result. This approach would modestly increase the reduction in SARS-CoV-2 transmission by avoiding false-negative test results and could facilitate more rapid quarantine of contacts. For example, if all symptomatic individuals were eligible for contact tracing (equivalent to 100% test sensitivity) and the delay to quarantine was just 12 h, the reduction in transmission would increase from 26% to 31%.

To avoid quarantine of large numbers of individuals when the incidence of infection is high, testing of contacts could be done and only those who test positive for SARS-CoV-2 infection put into isolation. The effectiveness of such a test-trace-test strategy is substantially lower than than of test and trace because of the high probability of false-negative results among contacts tested early in their infection (figure 2B). In fact, a 48–72 h delay in identifying and testing contacts is more effective than just a 24 h delay because the probability of a false-negative result decreases. At 80% coverage and with a 48 h delay in tracing and testing, the reduction in transmission from this strategy is 10% (95% UI −2 to 21), whereas with 50% coverage it is just 4% (−7 to 15). Negative effectiveness results from the release of symptomatic index cases from isolation following a false-negative test result.

**Figure 3:**
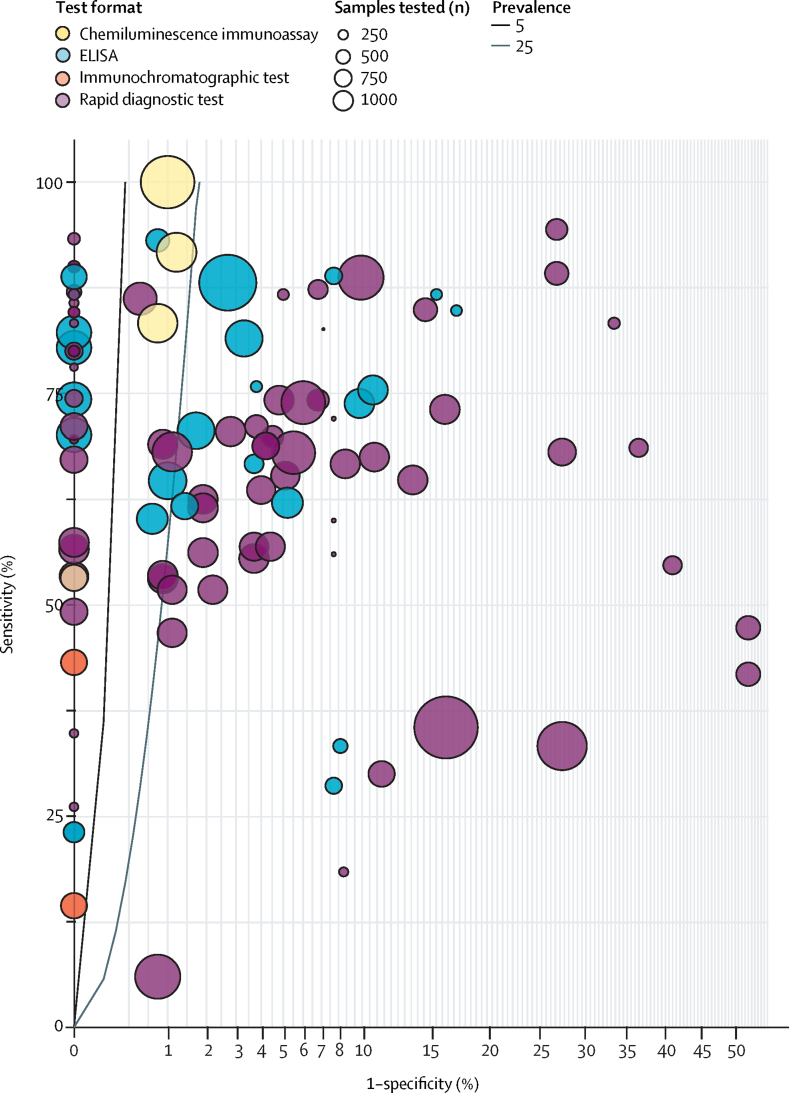
Sensitivity and specificity of currently available antibody tests The circles indicate the reported test performance for different test platforms, with the size of the circle proportional to the total number of control samples tested and the colour indicating the test format. The lines indicate thresholds for test performance required to provide a 95% probability of correctly predicting the presence of antibodies (positive predictive value) for a prevalence of 5% (black line) or 25% (grey line) among those tested. Tests to the left of these lines meet this performance standard, although further clinical sample testing is required to confirm their specificity. The x-axis shows 1-specificity plotted with a square root transform to better show the high specificity threshold required. Only tests for IgG, or IgG plus either IgM or IgA, are included. Data are from the Foundation for Innovative New Diagnostics.^12^

Antibody tests need to have high specificity to avoid false-positive results that would undermine the value of an antibody-based immunity passport. The performance of currently available tests compared with the standard required to provide a greater than 95% positive predictive value when prevalence is arbitrarily set at 5% or 25% is shown in figure 3. The specificity of antibody testing is around 90% or lower for rapid diagnostic tests but higher (eg, 95–99%) for laboratory-based ELISA and chemiluminescent assays.

## Discussion

As testing capacity increases, it is crucial that we use available SARS-CoV-2 tests not only to monitor the pandemic but also to directly contribute to the prevention of transmission. We estimate that weekly screening of health-care workers for infection through PCR testing would reduce their contribution to SARS-CoV-2 transmission by around one quarter on top of any reductions already achieved by self-isolation following onset of symptoms. The effectiveness of this strategy depends on the extent of transmission from presymptomatic and asymptomatic infected individuals and the timeliness of test results. Recent data from three UK hospitals that screened their staff found a prevalence of SARS-CoV-2 infection during April, 2020, of 2–3% in approximately 2000 health-care workers, all of whom were asymptomatic at the time of testing.^13^, ^14^, ^15^ In London, UK, data from March indicated a substantially higher prevalence of 7·1% (28 of 396) in the week of March 23,^15^ while seroprevalence in health-care workers in Birmingham, UK, on April 24, was 24·4% (126 of 516).^14^ It remains unclear how many of these infections were acquired from patients or from other health-care workers, although a high prevalence among staff who had no interaction with patients with COVID-19 in these studies suggests substantial transmission between health-care workers. More generally, nosocomial transmission has been an important feature of the pandemic, with UK data suggesting around 20% of patients admitted to hospital for COVID-19 acquired their infection in a hospital.^4^ Tests in residential care homes done during the same period also found a high prevalence of asymptomatic infection among staff.^16^ These and other groups with a high transmission risk and with frequent person-to-person contact, such as cashiers or teachers, might also therefore benefit from regular PCR testing during times of high SARS-CoV-2 incidence.

Detection of infected staff through regular screening or symptomatic testing also allows subsequent contact tracing at the workplace and implementation of infection control measures. Regular screening can also be complemented by mask wearing and other hygiene practices in communal areas where personal protective equipment would not typically be worn.

The number of tests required for regular screening of health-care workers will depend on the planned coverage. In England in 2019, there were around 35 000 National Health Service (NHS) staff working in intensive care, infectious disease, or respiratory medicine.^17^ Weekly testing of this group would require 5000 daily tests, considerably lower than the UK target of 200 000 daily tests by the end of May, 2020. Of course, NHS staff exposed to COVID-19 include other specialties and roles, and there has been considerable reassignment of NHS staff to care for patients with COVID-19, making the number at risk who would benefit from testing difficult to determine but likely to be considerably greater than 35 000.

Extending regular PCR screening, irrespective of symptoms, to the general population would be logistically impossible and inefficient.^6^ Instead, a test-and-trace strategy based on testing of symptomatic individuals and tracing the contacts of those with laboratory-confirmed SARS-CoV-2 infection is more appropriate. However, we estimate this strategy would at best prevent about 26% (95% UI 14–35) of transmission compared with self-isolation based on symptoms alone, depending on the extent of asymptomatic transmission, assuming 80% of symptomatic infections are reported, 80% of their contacts are traced and effectively quarantined (no onwards transmission), and that testing is done on the day of symptom onset with just a 24 h delay to test results and quarantine of contacts. The combined effectiveness of self-isolation based on symptoms (47% reduction in *R*) and a test-and-trace strategy (26% reduction in *R*) would be a 61% reduction in *R* based on these assumptions and our best estimates of the prevalence of asymptomatic infection and its contribution to SARS-CoV-2 transmission. If *R* in the absence of these interventions is greater than 2·5, as estimated for most European countries at the start of the pandemic and probably still the case given the relatively low prevalence of immunity (<10%), this strategy would be insufficient to achieve *R* lower than 1.^18^

A less ambitious test-and-trace approach or one with limited compliance would have a much smaller impact on SARS-CoV-2 transmission. For example, the probability of successful contact tracing depends on the ability to identify and follow-up contacts. However, contacts at risk of infection might be difficult to identify and could include not only direct interactions but also use of shared resources or spaces within a certain time period. Compliance with quarantine measures will depend on the level of trust in the government, individual perception of risk, economic incentives and disincentives, and the ability to impose quarantine on individuals. In the case of mobile phone applications (apps) for contact tracing, their effectiveness will depend on uptake of the app and the proportion of the population with a suitable mobile phone. It is quite possible that these considerations would result in low coverage (reporting of symptoms and identification of contacts) and limited effectiveness of test and trace.

There are also concerns about the speed with which cases can be detected and their contacts quarantined. In China, early in the epidemic the mean time from symptom onset to isolation of index cases was 4·6 days compared with 2·7 days for symptomatic contacts.^19^ Thus, although contact tracing reduced the time to isolation of infected individuals by 1·9 days, self-isolation began on average more than a week after infection, which would lead to minimal reduction in SARS-CoV-2 transmission according to our model (corresponding to a delay from test to trace and quarantine of about 5·5 days, since without a delay we expect quarantine of contacts on average 2·5 days after infection).

Contact tracing on the basis of symptoms alone is predicted to result in a modest increase in effectiveness compared with test and trace, assuming a similar proportion of contacts are quarantined. However, this strategy would result in unnecessary quarantine for a large number of people, particularly during the winter when respiratory viruses causing symptoms compatible with COVID-19 (eg, fever and cough) are common.^20^ Test and trace would result in fewer contacts requiring quarantine, although the number could still be substantial. For example, during the first week of test and trace implementation in England when incidence was relatively low (May 28 to June 3, 2020), 5407 (67%) of 8117 people testing positive had their contacts traced, with 26 985 contacts asked to quarantine themselves for 14 days.^21^ Testing of contacts and releasing those who test negative from quarantine would decrease the number in quarantine, but this approach substantially reduces the effectiveness of test and trace because of the high probability of false-negative test results in the first 3 days of infection.

Confirmation of SARS-CoV-2 infection through PCR testing could be used to confer an immunity passport on the basis of evidence of SARS-CoV-2 infection following self-isolation. Among those not tested by PCR, antibody testing at least 3–4 weeks after symptom onset could be used instead to determine immune status to SARS-CoV-2. It has been proposed that individuals with detectable antibodies could return to work, including to high-risk roles where exposure to infection is likely to be greatest. They could also be exempt from the need to self-isolate if a household member develops COVID-19 symptoms. However, there are a number of technical, logistic, and ethical challenges to the implementation of immunity passports. The first relates to the specificity of antibody testing. Poor specificity will result in non-immune individuals receiving an immunity passport and being potentially put at risk of infection. Conversely, using tests with poor sensitivity would compromise the effectiveness of the scheme and would result in individuals with acquired immunity being ineligible for an immunity passport. Sensitivity depends not only on the quality of the particular test, but also on antibody titre, which is lower following mild illness and in children and young adults than in older individuals.^22^

A second challenge for immunity passports is whether detectable antibody or PCR evidence of infection indicate protection against COVID-19, and if so, for how long. The initial rise in antibody titre 1–2 weeks after symptom onset is associated with clearance of SARS-CoV-2 infection, and passive transfer of antibodies from convalescent sera has been reported to improve outcomes in non-randomised trials.^23^, ^24^ However, the effectiveness and duration of protection against subsequent exposure is unknown, particularly against heterologous strains where weak cross-neutralising antibodies could result in enhanced pathology.^25^ Finally, there are concerns that the economic and personal benefits of an immunity passport to the general population would lead to fraud, and implementation of such a scheme is likely to face serious legal and ethical challenges related to discrimination based on immune status. Perhaps, instead of an immunity passport conferring specific privileges, evidence of immunity could be used by individuals to assess their COVID-19 risk and make their own informed decisions, particularly as our understanding of immunity to SARS-CoV-2 improves and assays become more reliable. Once vaccines become available, evidence of immunity is likely to be replaced by evidence of immunisation, with most (but not all) countries unlikely to discriminate on the basis of immune status.

There are limitations to our analysis, most notably the continued uncertainty around the contribution of asymptomatic infections to SARS-CoV-2 transmission, which we have addressed through the sensitivity analysis. Data on the implementation and impact of test-and-trace strategies and on the contribution of health-care workers to nosocomial transmission are also needed. As these strategies are adopted in the UK and more widely, it is important to collect high-quality data to improve models and investigate further refinements to testing strategies.

It is clear that PCR and antibody testing are required for surveillance of the COVID-19 pandemic and will play an important role in informing the lifting or re-imposing of various components of physical distancing interventions by allowing accurate estimates of *R* and identifying the extent of transmission. They can also play a direct role in prevention of SARS-CoV-2 transmission, with effectiveness strongly dependent on coverage and speed to results. Now is the time to invest in testing capacity, policy, and planning to maximise their contribution to the fight against COVID-19.
