# Supplementary material for: Comparison of molecular testing strategies for COVID-19 control: a mathematical modelling study
Source: Lancet Infect Dis. 2020 Dec;20(12):1381–9. doi: 10.1016/S1473-3099(20)30630-7 (PMC7434438; doi:10.1016/S1473-3099(20)30630-7)
Supplement: Supplementary appendix [file mmc1.pdf]

THE LANCET
Infectious Diseases

Supplementary appendix

This appendix formed part of the original submission and has been peer reviewed. We post it as supplied by the authors.

Supplement to: Grassly NC, Pons-Salort M, Parker EPK, et al. Comparison of molecular testing strategies for COVID-19 control: a mathematical modelling study. *Lancet Infect Dis* 2020; published online August 18. https://doi.org/10.1016/S1473-3099(20)30630-7.

Appendix: Role of molecular testing in COVID-19 control: a mathematical modelling study

Nicholas C Grassly DPhil[1], Margarita Pons-Salort PhD[1], Edward PK Parker PhD[2], Peter J White PhD[1], Neil M Ferguson DPhil[1] on behalf of the Imperial College COVID-19 Response Team[*]

[1] *MRC Centre for Global Infectious Disease Analysis, Department of Infectious Disease Epidemiology, Imperial College London, Norfolk Place, London W2 1PG*

[2] *The Vaccine Centre, Department of Clinical Research, Faculty of Infectious and Tropical Diseases, London School of Hygiene and Tropical Medicine, London, UK*

Includes:

Appendix Methods

Appendix Table 1

Appendix Figures 1-4

Appendix Methods

Mathematical Model

We assume infectiousness over time since infection follows a Weibull distribution, with a mean of 6 days consistent with published estimates of the serial interval for COVID-19.[1,2] This differs from SARS-CoV-2 shedding, which can be detected from about 3 days after infection (i.e. about 2 days before the onset of symptoms) and persists for up to 2-3 weeks at declining levels.[3-5] We denote infectiousness over time since infection τ by $\beta(\tau)$. The reproduction number R (expected number of secondary cases) is given by the integral of $\beta(\tau)$ over time since infection $R = \int_0^\infty \beta(\tau)d\tau$, and the generation time distribution $w(\tau)$ by unit normalisation such that $w(\tau) = \beta(\tau)/R$.[6] We assume the mean generation time is equivalent to the observed mean serial interval.

An infected individual may be symptomatic or asymptomatic and we define $\beta_a(\tau)$ and $\beta_s(\tau)$ respectively as their infectiousness over time since infection, which can be integrated to give their reproduction numbers R_a and R_s. Assuming random mixing with respect to symptoms, the overall reproduction number is given by $R = (1-s)R_a + sR_s$, where s is the proportion of individuals who develop symptoms.

Effectiveness of self-isolation

If infected individuals self-isolate following the onset of symptoms, then their infectiousness can be written as

$$\beta_{si}(\tau) = (1 - \Theta(\tau))\beta_s(\tau)$$

where $\Theta(\tau)$ is the proportion of these individuals who have developed symptoms and self-isolate by time τ. If we assume that individuals self-isolate immediately at onset of symptoms, then $\Theta(\tau)$ is the cumulative distribution function of the incubation period distribution, which we assume is gamma distributed with mean 5.5 days and variance 5.2 days as reported in Lauer et al. 2020.[7] We denote the reproduction number for symptomatic infected individuals with self-isolation as $R_{si} = \int_0^\infty \beta_{si}(\tau)\, d\tau$.

Average infectiousness for infected individuals when accounting for self-isolation is therefore

$$\beta(\tau) = (1-s)\beta_a(\tau) + s(1 - \Theta(\tau))\beta_s(\tau)$$

If the relative infectiousness of asymptomatic compared with symptomatic infections is r such that $\beta_a(\tau) = r\beta_s(\tau)$, then the proportion of transmission that is from symptomatic individuals is $s/(s + (1-s)r)$. This formulation means the effectiveness of self-isolation scales linearly with the proportion of transmission that is presymptomatic in the absence of self-isolation, which we estimate from $\Theta(\tau)$ and $\beta_s(\tau)$ to be about 40% (or 26% for a median serial interval of 8 vs. 6 days), consistent with available data.[8-10] If different estimates of this proportion become available, the effectiveness of self-isolation can therefore simply be scaled accordingly.

Effectiveness of regular screening

If PCR screening, for example of a HCW, identifies SARS-CoV-2 at time τ after infection, assuming this individual immediately self-isolates, then this would prevent a proportion of their transmission

$$p(\tau) = \int_{t=\tau}^{\infty} \frac{\beta(t)}{R}\, dt$$

In reality, self-isolation will not immediately follow sample collection. If we assume this takes x days, then the impact of screening at time t after infection is reduced from $p(\tau)$ to $p(\tau + x)$.

We assume a constant hazard of infection among HCWs over time, such that if we test them every T days the probability that they were infected at time t for $0 < t < T$ is simply $1/T$. If we had a test that was 100% sensitive from day zero after infection and could isolate HCWs immediately, then the proportion of new infections prevented by a strategy of screening every T days would be

$$\pi(T) = \int_{\tau=0}^{T} \frac{p(\tau)}{T}\, d\tau$$

For a test that has <100% sensitivity, we might miss infection the first time we screen. In this case

$$\pi(T) = \int_{\tau=0}^{T} \frac{S(\tau)\, p(\tau)}{T}\, d\tau$$

where $S(\tau)$ is the probability of detecting shedding of SARS-CoV-2 at time τ after infection. After multiple tests

$$\pi(T) = \int_{\tau=0}^{T} \frac{S(\tau)\, p(\tau)}{T}\, d\tau + \int_{\tau=0}^{T} \frac{(1 - S(\tau))S(T+\tau)\, p(T+\tau)}{T}\, d\tau + \cdots$$
$$\int_{\tau=0}^{T} \prod_{n=1}^{N-1} (1 - S((n-1)T + \tau))\, \frac{S(NT+\tau)\, p(NT+\tau)}{T}\, d\tau$$

where N is the maximum number of tests that would occur during a typical infectious period (and therefore depends on the interval T).

We explored a range of assumptions for $S(\tau)$ based on statistical synthesis of the literature. We identified 3 meta-analyses of test sensitivity over time since the onset of symptoms.[3-5] Only one attempted to estimate presymptomatic sensitivity,[4] but this was based on very limited data and used a cubic polynomial spline regression that did not match the data in the first few days after onset of symptoms. We therefore assumed presymptomatic sensitivity for days 1-4 post infection was proportional to infectiousness $\beta(\tau)$ with a proportionality constant that ensured the estimate on day 5 matched the empirical data from the day of onset of symptoms. For day 5 onwards, we used the estimates from Wikramanata (2020)[5] for sensitivity from the day of onset of symptoms, which fell between the estimates from the other two studies (Appendix Figure 1). We did not include differing test sensitivity by symptoms because estimates of the proportion of infections that are asymptomatic are largely based on PCR testing (in practice, this means that infections with very low viral load that may not be detected and are unlikely to contribute to transmission are not included) and because of uncertainty about the extent of viral shedding from asymptomatic compared with symptomatic individuals.[11-13]

Effectiveness of contact tracing and role of testing

To evaluate the effectiveness of contact tracing we consider the number of infections generated by the infected contacts of someone infected with SARS-CoV-2 (index case), which we denote by R_2 (the number of infections generated by a single infectious individual after two generations of transmission). R_2 will be the product of the number of infections generated by the index case (first generation of infections), which is given by R when symptomatic individuals self-isolate, and the number of infections generated by the contacts of the index case (second generation of infections), which we estimate here.

We can estimate R_2 in the context of contact tracing by considering symptomatic and asymptomatic infected individuals separately. We assume asymptomatic individuals are not the index case for contact investigations (i.e. their contacts will not be traced). The contribution of asymptomatic infected individuals to R_2 is therefore $(1 - s)R_a R$, where R is the contribution to transmission of their contacts (who are not traced). We assume symptomatic infected individuals are identified as index cases initiating contact tracing with probability u, and their contacts are traced with probability v. These probabilities relate to the effectiveness of identifying index cases and their contacts respectively, and will depend on the nature of the contact-tracing method (e.g. manual, app-based, etc.) and the definition of a contact. The contribution to R_2 of symptomatic infections who are not identified for contact tracing is $(1 - u)sR_{si}\, R$ (assuming the symptomatic index case self-isolates).

Finally, the contribution of symptomatic infected individuals where contact tracing is attempted depends on v and the time at which contacts are identified relative to their onset of infection. This depends on the time at which contact tracing is initiated relative to the onset of infection in the index case τ_i, which we assume is given by the distribution of the time to onset of symptoms $\theta(\tau_i)$, the number of secondary infections generated by the index case until that time, and the length of time these secondary infections are infected prior to their quarantine (illustrated in Appendix Figure 4). This gives the expected number of secondary cases after two generations from these symptomatic infected individuals identified for contact tracing as:

$$vsu \int_0^\infty \theta(\tau_i) \int_0^{\tau_i} \beta_s(\tau_2) \int_0^{\tau_i-\tau_2+q} \beta(\tau_q)\, d\tau_q\, d\tau_2\, d\tau_i + (1-v)suR_{si}R$$

where τ_q is the time of quarantine with respect to the onset of infection in the contact, τ_2 is the time of transmission from the index case relative to their onset of infection and q is the delay between initiating contact tracing and quarantine of the case. Summing these contributions and simplifying gives

$$R_2 = vsu \int_0^\infty \theta(\tau_i) \int_0^{\tau_i} \beta_s(\tau_2) \int_0^{\tau_i-\tau_2+q} \beta(\tau_q)\, d\tau_q\, d\tau_2\, d\tau_i + [s(1-uv)R_{si} + (1-s)R_a]R$$

The reproduction number for the second generation of transmission R_2' is simply R_2/R. In the absence of contact tracing and quarantine $\tau_i = \infty$ then $R_2 = R^2$ (and $R_2' = R$). We introduce some notation to simplify this expression. We write $R_2' = A + B$ where A is the contribution to transmission from traced contacts, and B the contribution to transmission from non-identified contacts:

$$\begin{aligned} R_2' &= A + B \\ A &= \frac{vsu}{R} \int_0^\infty \theta(\tau_i) \int_0^{\tau_i} \beta_s(\tau_2) \int_0^{\tau_i-\tau_2+q} \beta(\tau_q)\, d\tau_q\, d\tau_2\, d\tau_i \\ B &= s(1-uv)R_{si} + (1-s)R_a \end{aligned}$$

If symptomatic index cases only have their contacts traced if they test positive, then the probability of isolation and contact tracing at time τ_i after their onset of infection becomes $S_o\theta(\tau_i)$ where S_o is the sensitivity of the test on the day of onset of symptoms (assumed constant) and

$$\begin{aligned} A &= \frac{vsu}{R} \int_0^\infty S_o\, \theta(\tau_i) \int_0^{\tau_i} \beta_s(\tau_2) \int_0^{\tau_i-\tau_2+q} \beta(\tau_q)\, d\tau_q\, d\tau_2\, d\tau_i \\ B &= s(1-uvS_o)R_{si} + (1-s)R_a \end{aligned}$$

where for simplicity we ignore the contribution to transmission of false negative index cases after they receive their test results and exit self-isolation. This contribution is minimal since test sensitivity after symptom onset is a function of the quantity of virus being shed, but can result in a marginally higher R for 'test and trace' compared with self-isolation based on symptoms alone when sensitivity is low (therefore resulting in negative effectiveness of this strategy).

In the next (third) generation of infection, the number of infected individuals (R_3') will depend on whether contact tracing is performed for symptomatic contacts of the index case who test positive (i.e. whether contacts are themselves eligible for contact tracing). In addition, quarantine of successfully traced contacts will result in an altered (shorted) serial interval for any onwards transmission. Where the delay from test to trace is short (<24 hours) the contribution of traced contacts to transmission is relatively small $A/(A+B) < 15\%$ for coverage up to 80%. In this case, if we assume that traced symptomatic contacts are eligible as index cases for contact tracing and that the probability of successful identification as a contact (v) is independent of the probability of being identified for contact tracing (u), then we can ignore their altered serial duration and $R_3' \approx R_2'$ and by extension the reproduction number under a test-and-trace strategy is $R_{TT} \approx R_2'$. In reality u and v are likely to be correlated because of behavioural factors, or if contact tracing is implemented through a smartphone app. In this case, R_{TT} will be higher, depending on the magnitude of the correlation and the extent of transmission from untraced contacts, given by $s(1-uv)R_{si}$.

We can extend this model to consider testing of contacts and their release from quarantine if they test negative. In this case

$$\begin{aligned} A &= \frac{vsu}{R} \int_0^\infty S_o\, \theta(\tau_i) \int_0^{\tau_i} S(\tau_i - \tau_2 + q)\beta_s(\tau_2) \int_0^{\tau_i-\tau_2+q} \beta(\tau_q)\, d\tau_q\, d\tau_2\, d\tau_i \\ B &= vsu \int_0^\infty \theta(\tau_i) \int_0^{\tau_i} (1 - S(\tau_i - \tau_2 + q))\beta_s(\tau_2)\, d\tau_2\, d\tau_i + s(1-uvS_o)R_{si} + (1-s)R_a \end{aligned}$$

with the additional term in B representing the contribution to transmission in the second generation of contacts with false negative test results.

Appendix Table 1 Model parameter values.

Parameter	Symbol	Value (range)	Distribution for sensitivity analysis	Data source
Proportion of infections that are symptomatic (%)	s	67 (50-80)	uniform	1,14,15
Relative infectiousness of asymptomatic infections vs. symptomatic infections	r	0.5 (0.1-1.0)	uniform	11-13
Generation time distribution in absence of self-isolation	$w(\tau)$	Weibull (median 6 (5-8) days, shape 2.8)	uniform	Based on published serial intervals (1,2,10)
Incubation period distribution	$\theta(\tau)$	Gamma (mean 5.5 days, shape 5.8) (fixed)	NA	7
Sensitivity of SARS-CoV-2 test	$S(\tau)$	Empirical estimate (also consider lower sensitivity rescaled by 0.85)	NA	3-5
Sensitivity of SARS-CoV-2 at symptom onset	S_o	90% (80-95%)	uniform	3-5
Proportion of index infections identified for contact tracing (%)	u	0-100	NA	Varied in sensitivity analysis
Proportion of infected contacts successfully traced and isolated (%)	v	0-100	NA	Varied in sensitivity analysis
Time from onset of symptoms in index case to quarantine of their contacts (hours)	q	0-72	NA	Varied in sensitivity analysis

NA = not applicable

Appendix Figure 1 Estimated PCR test sensitivity over time since infection. The best estimates based on analysis of published data is shown (solid line) and the values used in a sensitivity analysis assuming 15% lower sensitivity (dashed line). See appendix methods for more details.

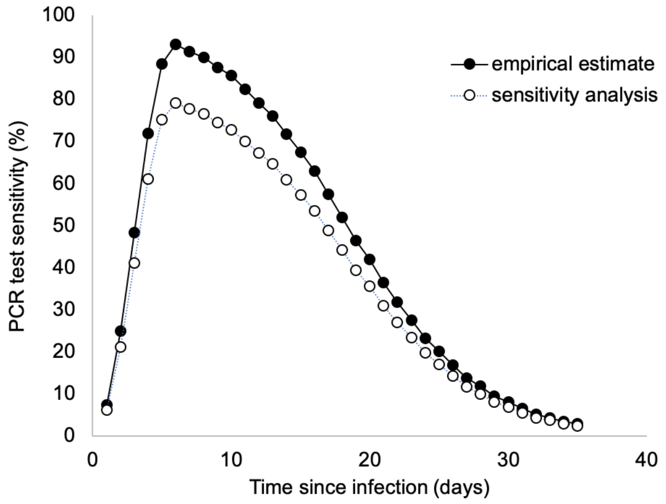

Appendix Figure 2 Sensitivity analysis of the effectiveness of regular screening of HCWs. In A) effectiveness (reduction in transmission from HCWs) is shown as a function of testing interval and timeliness of self-isolation for a median serial interval of 8 days instead of 6 days. In B) effectiveness of weekly screening with a 24-hour delay to results is shown as a function of the proportion of infections that are asymptomatic and their relative infectiousness compared with symptomatic infections (6 days serial interval). In C) the same effectiveness is shown but for test sensitivity over time since infection rescaled by 0.85 (i.e. 15% reduction).

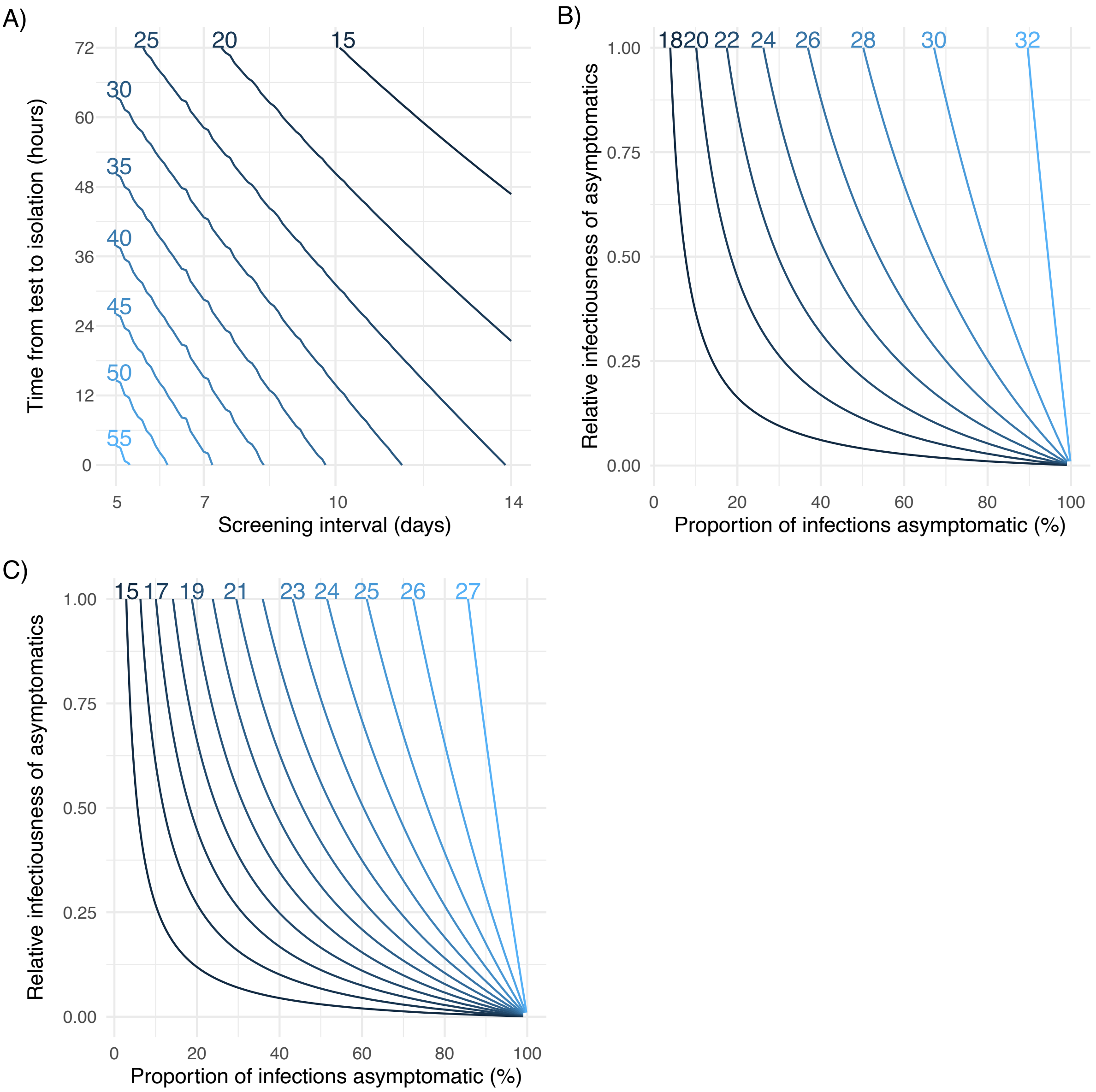

Appendix Figure 3 Sensitivity analysis of the effectiveness of test-and-trace. In A) effectiveness is shown as a function of the proportion of infections that are asymptomatic and their relative infectiousness compared with symptomatic infections. In B) effectiveness for different timeliness and coverage of test-and-trace is shown as for Figure 2A but with a median serial interval of 8 days rather than 6 days. Effectiveness is measured as the reduction in transmission from contacts of the index case as described in Figure 2.

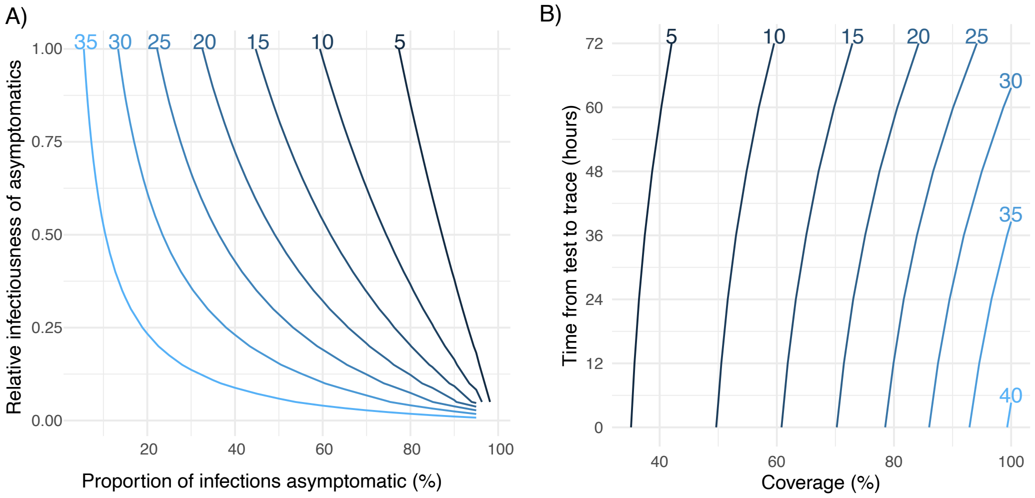

Appendix Figure 4 Illustration of the model of test-and-trace. Infectiousness over time is shown for the index case and for a single contact. Symbols correspond to those described in the model equations.

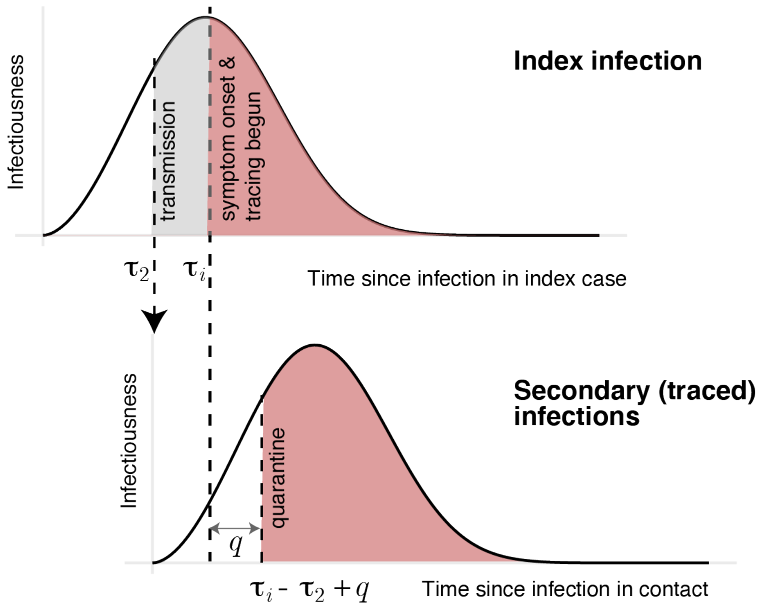

Appendix References

1. Bi Q, Wu Y, Mei S, et al. Epidemiology and transmission of COVID-19 in Shenzhen China: analysis of 391 cases and 1,286 of their close contacts. *Lancet Infect Dis* 2020; **20**: 911-9.
2. Ferretti L, Wymant C, Kendall M, et al. Quantifying SARS-CoV-2 transmission suggests epidemic control with digital contact tracing. *Science* 2020; **368**: eabb6936.
3. Borremans B, Gamble A, Prager KC, et al. Quantifying antibody kinetics and RNA shedding during early-phase SARS-CoV-2 infection. *medRxiv* 2020; *https://doi.org/10.1101/2020.05.15.20103275*.
4. Kucirka LM, Lauer SA, Laeyendecker O, Boon D, Lessler J. Variation in false-negative rate of reverse transcriptase poolymerase chain reaction–based SARS-CoV-2 tests by time since exposure. *Ann Intern Med* 2020: *https://doi.org/10.7326/M20-1495*.
5. Wikramaratna P, Paton RS, Ghafari M, Lourenco J. Estimating false-negative detection rate of SARS-CoV-2 by RT-PCR. *medRxiv* 2020: 2020.04.05.20053355.
6. Grassly NC, Fraser C. Mathematical models of infectious disease transmission. *Nat Rev Microbiol* 2008; **6**: 477-87.
7. Lauer SA, Grantz KH, Bi Q, et al. The incubation period of coronavirus disease 2019 (COVID-19) from publicly reported confirmed cases: estimation and application. *Annals Internal Med* 2020: *https://doi.org/10.7326/M20-0504*.
8. He X, Lau EHY, Wu P, et al. Temporal dynamics in viral shedding and transmissibility of COVID-19. *Nature Med* 2020; **26**: 672-5.
9. Du Z, Xu X, Wu Y, Wang L, Cowling BJ, Meyers LA. The serial interval of COVID-19 from publicly reported confirmed cases. *Emerg Infect Dis* 2020: 10.3201/eid2606.200357.
10. Ali ST, Wang L, Lau E, et al. Evolution of effective serial interval of SARS-CoV-2 by non-pharmaceutical interventions. Research Square preprint. *https://doi.org/10.21203/rs.3.rs-32486/v1* Accessed 15 June 2020.; 2020.
11. Liu Y, Yan L-M, Wan L, et al. Viral dynamics in mild and severe cases of COVID-19. *Lancet Infect Dis* 2020: 10.1016/S473-3099(20)30232-2.
12. Li R, Pei S, Chen B, et al. Substantial undocumented infection facilitates the rapid dissemination of novel coronavirus (SARS-CoV-2). *Science* 2020; **368**(6490): 489.
13. Cereda D, Tirani M, Rovida F, et al. The early phase of the COVID-19 outbreak in Lombardy, Italy. *arXiv* 2020: 2003.09320.
14. Mizumoto K, Kagaya K, Zarebski A, Chowell G. Estimating the asymptomatic proportion of coronavirus disease 2019 (COVID-19) cases on board the Diamond Princess cruise ship, Yokohama, Japan, 2020. *Eurosurveillance* 2020; **25**(10): 2000180.
15. Verity R, Okell LC, Dorigatti I, et al. Estimates of the severity of COVID-19 disease. *Lancet Infect Dis* 2020; **20**: 669-77.
